# Supplementary material for: Reconstructing the post-glacial spread of the sand fly Phlebotomus mascittii Grassi, 1908 (Diptera: Psychodidae) in Europe
Source: Commun Biol. 2023 Dec 8;6:1244. doi: 10.1038/s42003-023-05616-1 (PMC10709326; doi:10.1038/s42003-023-05616-1)
Supplement: Supplementary file 2 — Supplementary Information [file 42003_2023_5616_MOESM2_ESM.pdf]

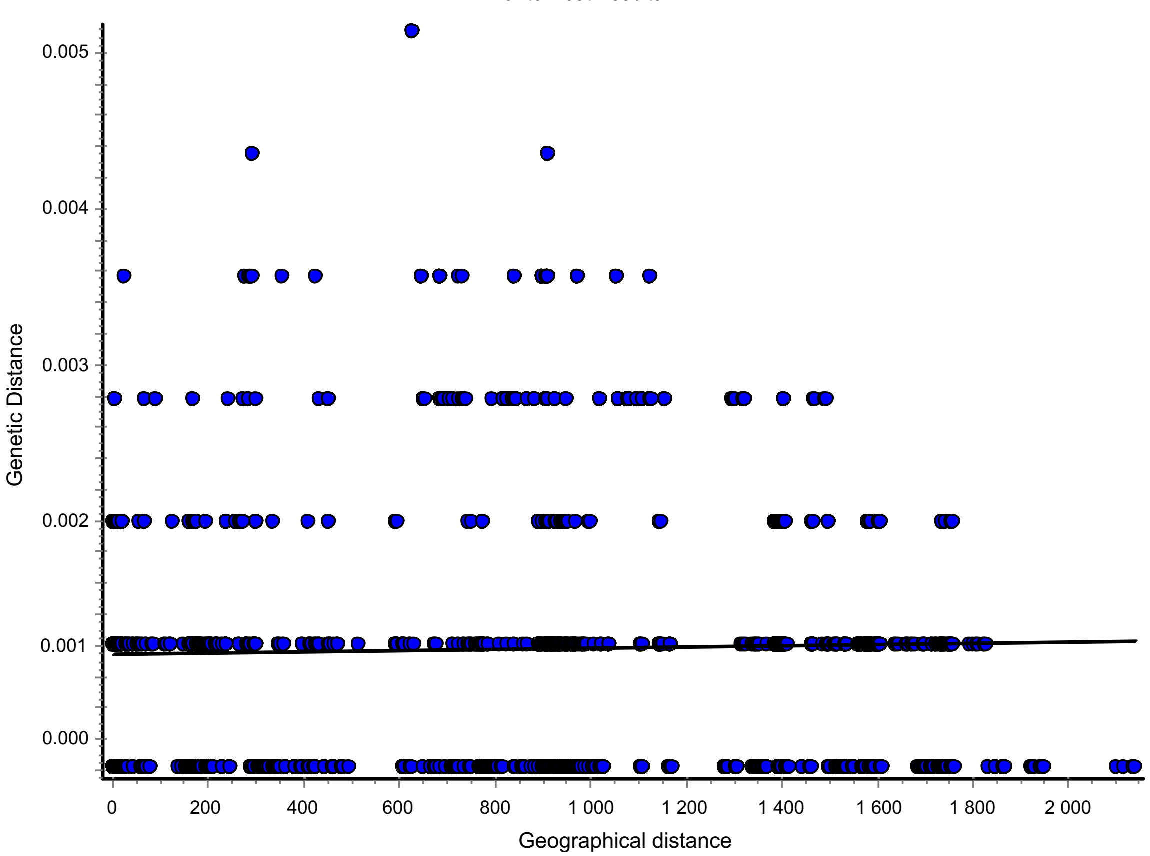
0.005
0.004
0.003
0.002
0.001
0.000
Genetic Distance
0
200
400
600
800
1 000
1 200
1 400
1 600
1 800
2 000
Geographical distance

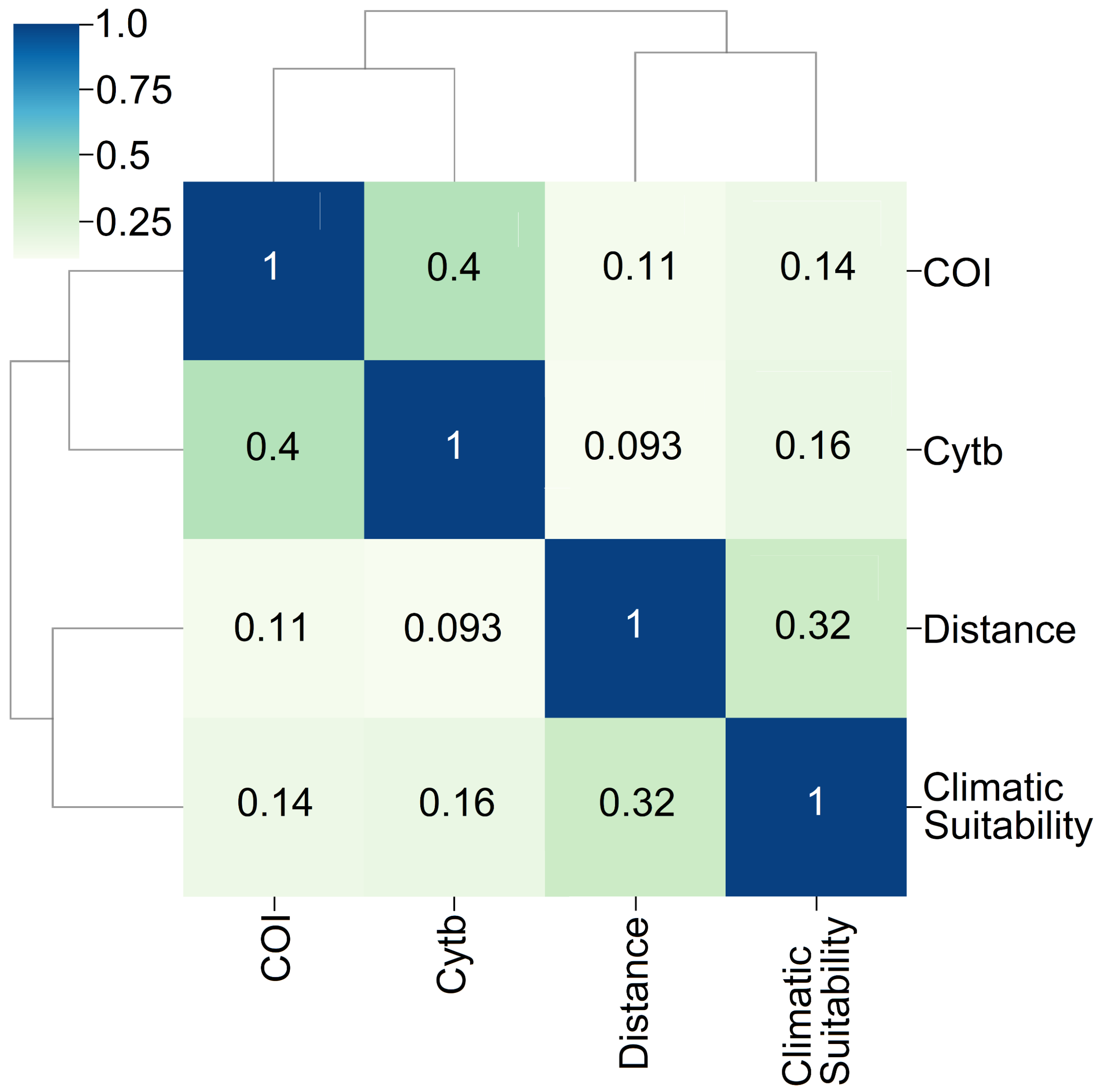

1.0
0.75
0.5
0.25
1
0.4
0.11
0.14
COI
0.4
1
0.093
0.16
Cytb
0.11
0.093
1
0.32
Distance
0.14
0.16
0.32
1
Climatic Suitability
COI
Cytb
Distance
Climatic Suitability

Supplementary Figure captions

File name: Supplementary Fig. 1.

Description: Inference of correlation between genetic and geographic distances of sampled individuals.

File name: Supplementary Fig. 2.

Description: Network similarity-based hierarchical clustering of the factors.

Supplementary Table 1. Uncorrected pairwise genetic COI distances (%) between identified haplotypes.

	**Haplotype**	**1**	**2**	**3**	**4**
**1**	COI_1	-			
**2**	COI_2	0.16	-		
**3**	COI_3	0.32	0.16	-	
**4**	COI_4	0.32	0.16	0.32	-

Supplementary Table 2. Uncorrected pairwise genetic Cytb distances (%) between identified haplotypes.

	**Haplotype**	**1**	**2**	**3**	**4**	**5**	**6**	**7**	**8**	**9**
**1**	Cytb_1	-								
**2**	Cytb_2	0.16	-							
**3**	Cytb_3	0.31	0.47	-						
**4**	Cytb_4	0.16	0.31	0.47	-					
**5**	Cytb_5	0.16	0.31	0.47	0.31	-				
**6**	Cytb_6	0.16	0.31	0.16	0.31	0.31	-			
**7**	Cytb_7	0.16	0.31	0.47	0.31	0.31	0.31	-		
**8**	Cytb_8	0.16	0.31	0.47	0.31	0.31	0.31	0.31	-	
**9**	Cytb_9	0.16	0.31	0.47	0.31	0.31	0.31	0.31	0.31	-

Supplementary Table 3. The used climatic variables in modelling.

Variable	Description	Unit
**bio1**	Mean Annual Temperature	(°C)
**bio2**	Mean Diurnal Range (Mean of monthly (max temp - min temp))	(°C)
**bio3**	Isothermality (bio2/bio7) (×100)	(°C)
**bio4**	Temperature Seasonality (standard deviation ×100)	(°C)
**bio5**	Max Temperature of Warmest Month	(°C)
**bio6**	Min Temperature of Coldest Month	(°C)
**bio7**	Temperature Annual Range (bio5-bio6)	(°C)
**bio8**	Mean Temperature of Wettest Quarter	(°C)
**bio9**	Mean Temperature of Driest Quarter	(°C)
**bio10**	Mean Temperature of Warmest Quarter	(°C)
**bio11**	Mean Temperature of Coldest Quarterx	(°C)
**bio12**	Annual Precipitation Sum	mm
**bio13**	Precipitation of Wettest Month	mm
**bio14**	Precipitation of Driest Month	mm
**bio15**	Precipitation Seasonality (Coefficient of Variation)	mm
**bio16**	Precipitation of Wettest Quarter	mm
**bio17**	Precipitation of Driest Quarter	mm
**bio18**	Precipitation of Warmest Quarter	mm
**bio19**	Precipitation of Coldest Quarter	mm

Supplementary Table 4. The distribution-limiting extrema of *Phlebotomus mascittii*.

Variable	min	max
**bio1**	7.9°C	16.7°C
**bio2**	3.7°C	8.8°C
**bio3**	1.9°C	3.3°C
**bio4**	4620°C	7785°C
**bio5**	214°C	302°C
**bio6**	-5.2°C	8.2°C
**bio7**	17.6°C	30.8°C
**bio8**	3.4°C	20.7°C
**bio9**	-0.5°C	24.8°C
**bio10**	17.2°C	25.8°C
**bio11**	-1.0°C	9.9°C
**bio12**	551 mm	1846 mm
**bio13**	65 mm	219 mm
**bio14**	10 mm	103 mm
**bio15**	10 mm	53 mm
**bio16**	195 mm	641 mm
**bio17**	39 mm	324 mm
**bio18**	49 mm	590 mm
**bio19**	99 mm	406 mm
