# Supplementary material for: Reconstructing the post-glacial spread of the sand fly Phlebotomus mascittii Grassi, 1908 (Diptera: Psychodidae) in Europe
Source: Commun Biol. 2023 Dec 8;6:1244. doi: 10.1038/s42003-023-05616-1 (PMC10709326; doi:10.1038/s42003-023-05616-1)
Supplement: Supplementary file 3 — Description of Additional Supplementary Files [file 42003_2023_5616_MOESM3_ESM.docx]

**Description of Additional Supplementary Files**

**File name:** Supplementary Data 1.

**Description:** *Phlebotomus mascittii* specimens analyzed in this study. References of first detection at locations are given.

**File name:** Supplementary Data 2.

**Description:** Raw data for the mismatch distribution, the Baysian skyline plot and isolation by distance.

**File name:** Supplementary Data 3.

**Description:** Protein alignment of analyzed COI sequences.

**File name:** Supplementary Data 4.

**Description:** Protein alignment of analyzed Cytb sequences.

**File name:** Supplementary Data 5.

**Description:** Script of the applied equation in the modelling of climatic suitability values of *Phlebotomus mascittii*.
