# Supplementary material for: Reconstructing the post-glacial spread of the sand fly Phlebotomus mascittii Grassi, 1908 (Diptera: Psychodidae) in Europe
Source: Commun Biol. 2023 Dec 8;6:1244. doi: 10.1038/s42003-023-05616-1 (PMC10709326; doi:10.1038/s42003-023-05616-1)
Supplement: Supplementary file 8 — Supplementary Data 5 [file 42003_2023_5616_MOESM8_ESM.pdf]

Supplementary Data 5. Script of the applied equation in the modelling of climatic suitability values of *Phlebotomus mascittii*.

("bio_1@1" >= 79) + ("bio_1@1" <= 167) + ("bio_2@1" >= 37) + ("bio_2@1" <=88) + ("bio_3@1" >= 19) + ("bio_3@1" <= 33) + ("bio_4@1" >= 4620) + ("bio_4@1" <= 7785) + ("bio_5@1" >= 214) + ("bio_5@1" <= 302) + ("bio_6@1" >= -52) + ("bio_6@1" <= 82) + ("bio_7@1" >= 176) + ("bio_7@1" <= 308) + ("bio_8@1" >= 34) + ("bio_8@1" <= 207) + ("bio_9@1" >= -5) + ("bio_9@1" <= 248) + ("bio_10@1" >= 172) + ("bio_10@1" <= 258) + ("bio_11@1" >= -10) + ("bio_11@1" <= 99) + ("bio_12@1" >= 551) + ("bio_12@1" <= 1846) + ("bio_13@1" >= 65) + ("bio_13@1" <= 219) + ("bio_14@1" >= 10) + ("bio_14@1" <= 103) + ("bio_15@1" >= 10) + ("bio_15@1" <= 53) + ("bio_16@1" >= 195) + ("bio_16@1" <= 641) + ("bio_17@1" >= 39) + ("bio_17@1" <= 324) + ("bio_18@1" >= 49) + ("bio_18@1" <= 590) + ("bio_19@1" >= 99) + ("bio_19@1" <= 406)

For the explanation of the variables see Supplementary Tables 3, 4.
