# Supplementary material for: Reconstructing the post-glacial spread of the sand fly Phlebotomus mascittii Grassi, 1908 (Diptera: Psychodidae) in Europe
Source: Commun Biol. 2023 Dec 8;6:1244. doi: 10.1038/s42003-023-05616-1 (PMC10709326; doi:10.1038/s42003-023-05616-1)
Supplement: Supplementary file 9 — Reporting Summary [file 42003_2023_5616_MOESM9_ESM.pdf]

nature portfolio

Corresponding author(s): Attila Trájer

Last updated by author(s): Nov 8, 2023

Reporting Summary

Nature Portfolio wishes to improve the reproducibility of the work that we publish. This form provides structure for consistency and transparency in reporting. For further information on Nature Portfolio policies, see our Editorial Policies and the Editorial Policy Checklist.

Statistics

For all statistical analyses, confirm that the following items are present in the figure legend, table legend, main text, or Methods section.

n/a	Confirmed	
☐	☒	The exact sample size (*n*) for each experimental group/condition, given as a discrete number and unit of measurement
☒	☐	A statement on whether measurements were taken from distinct samples or whether the same sample was measured repeatedly
☒	☐	The statistical test(s) used AND whether they are one- or two-sided<br>*Only common tests should be described solely by name; describe more complex techniques in the Methods section.*
☒	☐	A description of all covariates tested
☒	☐	A description of any assumptions or corrections, such as tests of normality and adjustment for multiple comparisons
☒	☐	A full description of the statistical parameters including central tendency (e.g. means) or other basic estimates (e.g. regression coefficient) AND variation (e.g. standard deviation) or associated estimates of uncertainty (e.g. confidence intervals)
☒	☐	For null hypothesis testing, the test statistic (e.g. F, t, r) with confidence intervals, effect sizes, degrees of freedom and P value noted<br>*Give P values as exact values whenever suitable.*
☐	☒	For Bayesian analysis, information on the choice of priors and Markov chain Monte Carlo settings
☒	☐	For hierarchical and complex designs, identification of the appropriate level for tests and full reporting of outcomes
☒	☐	Estimates of effect sizes (e.g. Cohen's *d*, Pearson's *r*), indicating how they were calculated

Our web collection on statistics for biologists contains articles on many of the points above.

Software and code

Policy information about availability of computer code

Data collection	No software was used for data collection. Analyzed data comprises DNA samples obtained from sand fly specimens that were caught during entomological surveys with standard light traps.
Data analysis	1. Sequence data was analyzed and edited with GeneDoc 2.7.0 and aligned using ClustalX (freewares). Haplotypes were calculated using DNAsp v. 5 and Popart for inferring the networks. Testing for signals of population expansion was inferred by using Arlequin v3.11 and BEAST 2.7.1 for a Bayesian coalescent approach (all freewares).<br>2. Maps were created in QGIS, the network was created using Gephi 0.10, heatmap was created in python.

For manuscripts utilizing custom algorithms or software that are central to the research but not yet described in published literature, software must be made available to editors and reviewers. We strongly encourage code deposition in a community repository (e.g. GitHub). See the Nature Portfolio guidelines for submitting code & software for further information.

Data

Policy information about availability of data

All manuscripts must include a data availability statement. This statement should provide the following information, where applicable:

- Accession codes, unique identifiers, or web links for publicly available datasets
- A description of any restrictions on data availability
- For clinical datasets or third party data, please ensure that the statement adheres to our policy

All the data supporting the findings of this study are available within the article and its Supplementary Information files. All DNA sequence data was uploaded to GenBank, and accession numbers are given in Supplementary Table 1. Additional information and relevant data are available from the corresponding author upon request.

Research involving human participants, their data, or biological material

Policy information about studies with human participants or human data. See also policy information about sex, gender (identity/presentation), and sexual orientation and race, ethnicity and racism.

Reporting on sex and gender	No sex based analysis was performed. We analyzed data of insect DNA, sand flies in particular, and inferred post-glacial spread based on genetic and climate modelling data, thus, discrimination between sexes was not necessary.
Reporting on race, ethnicity, or other socially relevant groupings	We analyzed insect samples, thus, no reporting on race, ethnicity or socially relevant grouping was done.
Population characteristics	We did not include human (data) in the analyses.
Recruitment	No human data analyzed and thus no recruitment necessary.
Ethics oversight	No human data analyzed.

Note that full information on the approval of the study protocol must also be provided in the manuscript.

Field-specific reporting

Please select the one below that is the best fit for your research. If you are not sure, read the appropriate sections before making your selection.

☐ Life sciences ☐ Behavioural & social sciences ☒ Ecological, evolutionary & environmental sciences

For a reference copy of the document with all sections, see nature.com/documents/nr-reporting-summary-flat.pdf

Ecological, evolutionary & environmental sciences study design

All studies must disclose on these points even when the disclosure is negative.

Study description	In this study, we performed a combined approach using genetic data and climate modelling to reveal glacial refugia and post-glacial spread of Ph. mascittii, the predominant sand fly species in Central Europe. In principal, all currently available data was included in our study.
Research sample	Research samples comprise Phlebotomus mascittii (Diptera: Psychodidae: Phlebotominae) sequences. These were obtained from either whole specimens (subsequent DNA isolation), provided DNA, or sequences obtained from GenBank. Ph. mascittii is widely distributed in Europe, however, low numbers are caught at positive sights. Therefore, our approach was to include as many specimens as possible, no matter where they originated from. The collection of samples comprises DNA sequences obtained from specimens originating from Spain, France, Corsica, Belgium, Germany, Italy, Slovenia, Austria, Bosnia, and Serbia. All available data was used from the countries. Available sequences of Ph. mascittii were obtained from GenBank.
Sampling strategy	Samples of Ph. mascittii are limited and were collected over several years, thus, all acquired data was used. For this reason, no sample size calculations were performed.
Data collection	Data collection was done by entomological fieldwork. Surveys were undertaken in various countries performed by respective experts in the countries. In principal, all samples (adult sand flies) were collected by standardized methods using CDC light traps. Subsequently, sand flies were identified by the experts and either whole specimens or DNA were provided for this study. We confirmed identity for all included samples by molecular barcoding. Information on trapping site, species and sex was recorded and provided.
Timing and spatial scale	Sand fly specimens were obtained during various entomological surveys dating from the year 2005 to 2021. The surveys were performed by different researchers in the respective countries. In our manuscript we provide a supplementary table 1 that comprises

	all used sequences in our study. We state vouchers, exact locations, haplotypes as well as references for the data collection that include the year of trapping.
Data exclusions	No data were excluded from the analysis. The nature of the sampled organism requires the usage of all available data, as specimens are limited. To grant high quality of all used data, DNA sequences of all specimens were obtained in both directions, aligned and consensus sequences were generated. In addition, protein alignments were checked for internal stop codons.
Reproducibility	All attempts to repeat experiments were successful. To grant full reproducibility, all generated sequences were deposited in GenBank and formulas for climate modelling are presented in the manuscipt.
Randomization	Randomization was not performed as all available data was used in our study.
Blinding	No blinding of data applied in our study. Our dataset does not comprise any sensitive data (e.g. human, animal study). We present a dataset solely comprising insect data, thus, no blinding was needed.

Did the study involve field work? ☒ Yes ☐ No

Field work, collection and transport

Field conditions	Data was collected during various fieldwork surveys, in different countries and different years. However, the season was always the same (June to September) and the method of collection was always standardized light trapping. This is the gold standard for sand fly trapping. Sand fly trapping is usually performed during summer month as activity is highest and rainfall is low. For our study, the timing of trapping is not relevant as we are not analyzing seasonality or other biological parameters. We only analyze DNA sequences of Ph. mascittii, which are independent from the season.
Location	Samplings were performed in different countries. We included sequences of specimens originating from 10 different countries: Spain, France, Corsica, Belgium, Germany, Italy, Slovenia, Austria, Bosnia, and Serbia. The exact locations of all used specimens are provided in supplementary table 1 in our study.
Access & import/export	For all entomological surveys, experts of the respective countries performed trappings. Sand flies are not protected species and, thus, no permission to perform trappings is required. When trapping at private properties, informed verbal consent of all homeowners was obtained to get permission to trap at their properties. For data collection of this study, either whole specimens, DNA or sequences were sent to Vienna for analyses. Whole sand fly specimens in 70% ethanol as well as DNA samples are not restricted and were shipped to Austria without restrictions.
Disturbance	Sand fly specimens were collected with standardized light traps during the night, no disturbance is caused by this method.

Reporting for specific materials, systems and methods

We require information from authors about some types of materials, experimental systems and methods used in many studies. Here, indicate whether each material, system or method listed is relevant to your study. If you are not sure if a list item applies to your research, read the appropriate section before selecting a response.

Materials & experimental systems

n/a	Involved in the study
☒	☐ Antibodies
☒	☐ Eukaryotic cell lines
☒	☐ Palaeontology and archaeology
☒	☐ Animals and other organisms
☒	☐ Clinical data
☒	☐ Dual use research of concern
☒	☐ Plants

Methods

n/a	Involved in the study
☒	☐ ChIP-seq
☒	☐ Flow cytometry
☒	☐ MRI-based neuroimaging
